# Automation and Application of a Direct-Current Plasma Emission Spectrometer

**DOI:** 10.6028/jres.093.118

**Published:** 1988-06-01

**Authors:** M. S. Epstein, R. E. Jenkins, K. S. Epler, T. C. O’Haver

**Affiliations:** Inorganic Analytical Research Division, National Bureau of Standards, Gaithersburg, MD 20899; Rice University, Houston, TX 77251; Department of Chemistry, University of Maryland, College Park, MD 20742

The direct-current plasma (DCP) coupled to an echelle spectrometer has been used for over 6 years in our laboratory as one of several independent methods for the certification of Standard Reference Materials (SRMs). The demanding certification process requires maximum performance from an analytical method as well as a good understanding of the method capabilities and limitations. We have made a number of modifications to the conventional DCP spectrometer to improve accuracy, precision, and analysis speed. Figure 1 is a schematic diagram of the entire DCP spectrometric system with all modifications and enhancements. This paper discusses these modifications and their application to a number of analytical problems.

## Conversion from Unmodulated to Wavelength- Modulated Detection

The advantages of wavelength-modulated detection over unmodulated detection schemes have been well-documented in the literature [1–3]. Signal-to-noise ratio (SNR) enhancement in the presence of background emission flicker noise, reduction of baseline drift, nulling of line spectral interferences and rapid background correction are some of the advantages of wavelength-modulated detection. Unmodulated detection is superior when spectral resolution is critical or when the limiting system noise is detector shot-noise. The DCP spectrometer and detection system were modified so that either unmodulated or wavelength-modulated schemes could be employed.

## Reduction of Spectrometer Wavelength Drift

Severe nonlinear drift of emission intensity was a recurrent problem with one of our two DCP/echelle emission spectrometers. The drift was a result of the extreme sensitivity of the spectrometer to thermal variations and vibration. Two approaches were taken to reduce the spectrometer drift. The first involved thermal regulation of the spectrometer baseplate at a temperature higher than attained during normal operation, and vibrational isolation of the spectrometer. These actions significantly reduced both long- and short-term intensity fluctuations.

Since the major heat source affecting the spectrometer was the plasma, another approach was removal of the plasma from contact with the spectrometer baseplate. The plasma was housed in a separate vented enclosure, and an optical rail with appropriate focusing optics was positioned between the spectrometer and the plasma. This configuration also effectively eliminated wavelength and order drift as major sources of intensity drift.

## Automation of Sample Introduction, Data Collection, and Processing

**Figure 2:**
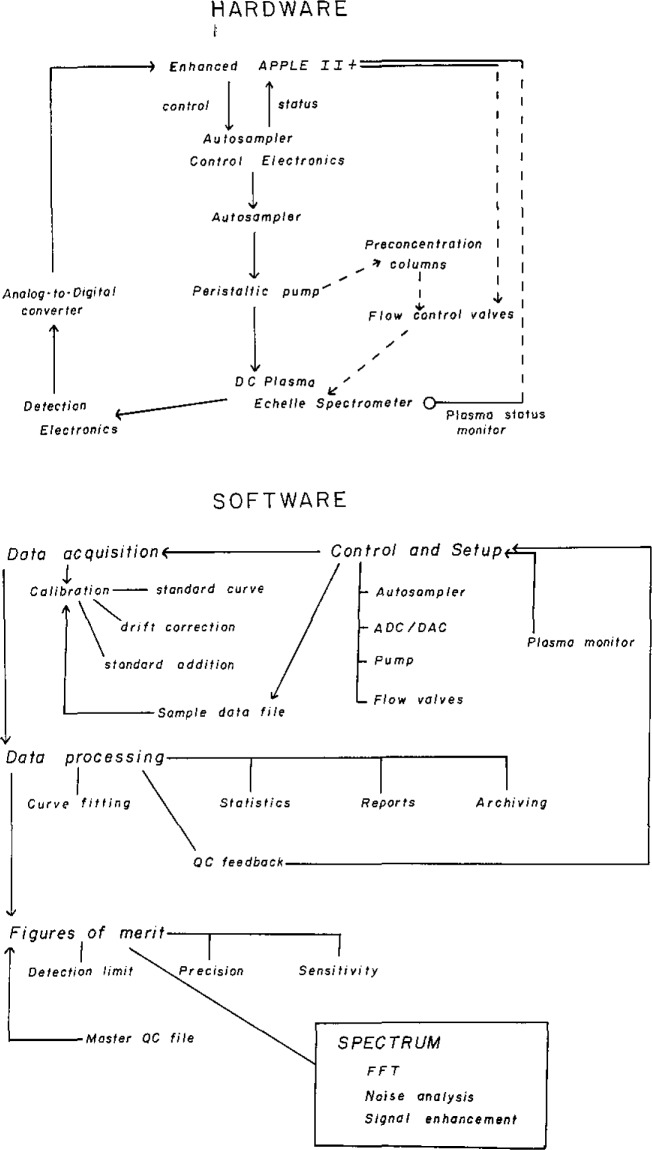
Software and hardware flow diagrams for the DCP spectrometric system. Dashed lines in the hardware diagram indicate components that are not yet automated.

An enhanced Apple II+ microcomputer (3.5 MHz 65C02 processor) coupled to an autosampler and controlled by a BASIC program was used to automate the DCP spectrometer operations. Figure 2 illustrates both the hardware and software design of the system. The autosampler was controlled through the three game control annunciator outputs of the Apple II+ and autosampler status was monitored through two game control pushbutton inputs. The three annunciator outputs were converted to five control states needed to program the autosampler by a binary-to-BCD converter. Voltage output from the spectrometer, either from a current-to-voltage converter (unmodulated system) or from a lock-in amplifier (wavelength-modulation system), was converted to digital data by a 12-bit ADC and processed by the BASIC program. Standard and sample weights and dilution factors are entered into the program at the start of an analysis and a report of sample concentrations is generated at the completion. Options to apply drift correction or to use a standard addition correction are also included in the software.

## Removal of Interfering Species Using Ion-Exchange Chromatography

The most straightforward approach to eliminate matrix-induced interferences, and the one that requires the least number of compromises, is the complete separation of analyte from interfering species. When chromatographic methods are directly coupled to spectrometric methods for online separations, sensitivity and accuracy may be further enhanced by preconcentration of the analyte on the chromatographic column.

We have applied an anion exchange method using an activated alumina (acid form) column for on-line preconcentration of phosphorus. Activated alumina has been found by several researchers [4,5] to be a useful column packing material for adsorption of oxyanions. The column is quite useful for the determination of phosphorus in acid digests of iron and copper-based alloys, since the direct DCP determination is complicated by iron and copper spectral interferences. Phosphorus is retained on the column while iron and copper are not retained and elute with a water wash of the column. With preconcentration times of 30 minutes, phosphorus detection limits are 20 times better than obtained using continuous aspiration. A detection limit of 10 ng/mL is obtained under these conditions, corresponding to a detection limit of 0.1 µg/g in the metal alloy. Results from the determination of phosphorus in several SRMs, as shown in table 1, were in good agreement with certified values or other independent techniques.

## Figures and Tables

**Figure 1:**
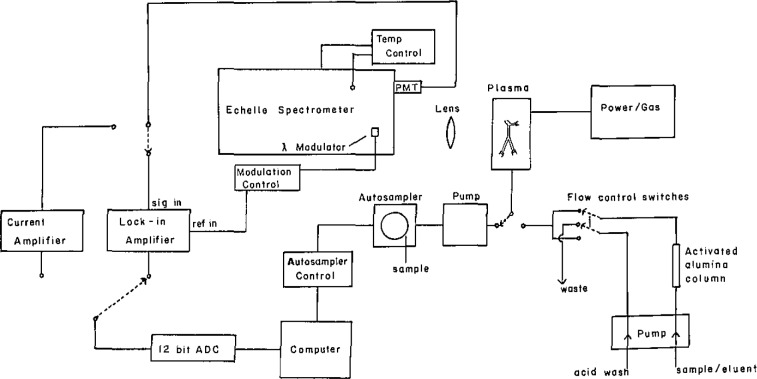
Schematic diagram showing all components of the DCP spectrometric system.

**Table 1:** Determination of phosphorus in standard reference materials using preconcentration on an activated alumina column

Sample	P, µg/g^a^	Other values	Method^b^
SRM 1252			
Phosphorized copper	84±8	85±2	Molybdivanadophosphoric acid method (ASTM E-62) [6]
SRM 875			
Cupro-nickel	21.4±1.8	20±5	Certified value
SRM 1767			
Low-alloy steel	37 ± 1	39±2	Modified ASTM 350–84 heteropolymolybdate procedure [7]
SRM 365			
Electrolytic iron	15±1	12±2	Modified ASTM 350–84 heteropolymolybdate procedure [7]
		14±2	Molecular absorption spectrometry [8]
		12.5	Indirect ICP method [8]
		25±5	Certified value

a Uncertainty expressed as 95% confidence limits.

b Values obtained by other techniques or certified values.
